# Severity of acute gastrointestinal injury grade is a predictor of all-cause mortality in critically ill patients: a multicenter, prospective, observational study

**DOI:** 10.1186/s13054-017-1780-4

**Published:** 2017-07-14

**Authors:** Bangchuan Hu, Renhua Sun, Aiping Wu, Yin Ni, Jingquan Liu, Feng Guo, Lijun Ying, Guoping Ge, Aijun Ding, Yunchao Shi, Changwen Liu, Lei Xu, Ronglin Jiang, Jun Lu, Ronghai Lin, Yannan Zhu, Weidong Wu, Bo Xie

**Affiliations:** 1 ICU, Zhejiang Provincial People’s Hospital, 158 Shangtang Road, Hangzhou, 310014 China; 2 ICU, Sir Run Run Shaw Hospital, Zhejiang University School of Medicine, 3 East Qingchun Road, Hangzhou, Zhejiang China; 3 ICU, Shaoxing People’s Hospital, Zhongxing North Road, Shaoxing, 312000 China; 4 ICU, Jinhua People’s Hospital, 228 Xinhua Street, Jinhua, 321000 China; 5 ICU, The Third Affiliated Hospital of Zhejiang Chinese Medical University, 219 Moganshan Road, Hangzhou, 310014 China; 6 ICU, The First Hospital of Jiaxing, 529 Hexin South Road, Jiaxing, 314000 China; 7 ICU, Hangzhou First People’s Hospital, 261 Huansha Road, Hangzhou, 310006 China; 8 ICU, Ningbo Medical Treatment Center, Lihuili Hospital, 57 Xingning Road, Ningbo, 315000 China; 9 ICU, The First Affiliated Hospital of Zhejiang Chinese Medical University, 54 Youdian Road, Hangzhou, 310006 China; 10 ICU, The Second Affiliated Hospital of Zhejiang Chinese Medical University, 318 Chaowang Road, Hangzhou, 310005 China; 11 ICU, Taizhou Hospital of Zhejiang Province, 150 Ziyang Old Street, Linhai, 317000 China; 12 ICU, Zhuji People’s Hospital of Zhejiang Province, 9 Jianming Road, Shaoxin, China; 13 ICU, The Central Hospital of Lishui City, 15 Dazhong Street, Lishui, 323000 China; 14 ICU, Huzhou Central Hospital, 198 Hongqi Road, Huzhou, 313003 China

**Keywords:** Critically ill patients, Acute gastrointestinal injury, Feeding intolerance, Mortality

## Abstract

**Background:**

In 2012, the European Society of Intensive Care Medicine proposed a definition for acute gastrointestinal injury (AGI) based on current medical evidence and expert opinion. The aim of the present study was to evaluate the feasibility of using the current AGI grading system and to investigate the association between AGI severity grades with clinical outcome in critically ill patients.

**Methods:**

Adult patients at 14 general intensive care units (ICUs) with an expected ICU stay ≥24 h were prospectively studied. The AGI grade was assessed daily on the basis of gastrointestinal (GI) symptoms, intra-abdominal pressures, and feeding intolerance (FI) in the first week of admission to the ICU.

**Results:**

Among the 550 patients enrolled, 456 patients (82.9%) received mechanical ventilation, and 470 patients were identified for AGI. The distribution of the global AGI grade was 24.5% with grade I, 49.4% with grade II, 20.6% with grade III, and 5.5% with grade IV. AGI grading was positively correlated with 28- and 60-day mortality (*P* < 0.0001). Univariate Cox regression analysis showed that age, sepsis, diabetes mellitus, coronary artery disease, the use of vasoactive drugs, serum creatinine and lactate levels, mechanical ventilation, Acute Physiology and Chronic Health Evaluation II (APACHE II) score, and the global AGI grade were significantly (*P* ≤ 0.02) associated with 60-day mortality. In a multivariate analysis including these variables, diabetes mellitus (HR 1.43, 95% CI 1.03–1.87; *P* = 0.05), the use of vasoactive drugs (HR 1.56, 95% CI 1.12–2.11; *P* = 0.01), serum lactate (HR 1.15, 95% CI 1.06–1.24; *P* = 0.03), global AGI grade (HR 1.65, 95% CI 1.28–2.12; *P* = 0.008), and APACHE II score (HR 1.04, 95% CI 1.02–1.06; *P <* 0.001) were independently associated with 60-day mortality. In a subgroup analysis of 402 patients with 7-day survival, in addition to clinical predictors and the AGI grade on the first day of ICU stay, FI within the first week of ICU stay had an independent and incremental prognostic value for 60-day mortality (χ^2^ = 41.9 vs. 52.2, *P* = 0.007).

**Conclusions:**

The AGI grading scheme is useful for identifying the severity of GI dysfunction and could be used as a predictor of impaired outcomes. In addition, these results support the hypothesis that persistent FI within the first week of ICU stay is an independent determinant for mortality.

**Trial registration:**

Chinese Clinical Trial Registry identifier: ChiCTR-OCS-13003824. Registered on 29 September 2013.

**Electronic supplementary material:**

The online version of this article (doi:10.1186/s13054-017-1780-4) contains supplementary material, which is available to authorized users.

## Background

Gastrointestinal (GI) problems in critically ill patients are common and associated with unfavorable outcomes [1, 2]. The GI system is considered critical to the development of multiple organ failure (MOF), with bacterial translocation in intensive care unit (ICU) patients supporting the concept of the gut having a role in MOF [3, 4]. However, there is no objective and clinically relevant definition of GI dysfunction in critically ill patients. In a recent consensus statement, the working group on abdominal problems of the European Society of Intensive Care Medicine (ESICM) proposed a grading system and treatment of acute gastrointestinal injury (AGI) based on current medical evidence and expert opinion [5]. Because the current AGI grading system is somewhat complicated and not based on objective variables, additional studies are needed to validate the clinical feasibility of the recommendations for grading GI function. In addition, the associations between AGI grade, the severity of GI dysfunction, and adverse outcome remain to be elucidated.

Feeding intolerance (FI) is a marker of GI dysfunction [1], but due to the lack of a consistent definition of FI, the prevalence of FI has been reported to vary remarkably among studies [6, 7]. Despite the problems with definition, FI has been suggested to occur frequently and to be associated with adverse outcomes in critically ill patients [1, 6]. Nevertheless, the exact role of FI with regard to mortality remains controversial [8–11]. It is uncertain whether FI simply represents an epiphenomenon of illness severity or harm from inadequate enteral nutrition (EN) and/or the use of parenteral nutrition [12]. Therefore, the aims of the present study were to investigate whether the current AGI grading system could be used to objectively evaluate the severity of GI dysfunction and its association with clinical outcome and to further explore whether FI defined on the basis of the recommendation of the ESICM has independent and incremental prognostic significance in critically ill patients.

## Methods

### Study design

This prospective, observational, multicenter study was conducted in 14 general ICUs of Zhejiang Province from 1 March to 31 August 2014. The patients were recruited from 1 March to 30 April 2014. The last follow-up was completed on 30 June 2014; the remaining 2 months of the study were for data management and analysis. To be eligible, ICUs had to have a minimum of 12 beds and have a dedicated senior physician with adequate knowledge of clinical nutrition as well as a dietitian responsible for data collection. The AGI grade was assessed daily according to the recommendation of the ESICM grading system during the first week of the subject’s ICU stay. This system is based mainly on GI symptoms and intra-abdominal pressure (IAP) on days 1–3 of ICU admission, and it is concomitantly combined with FI and organ dysfunction on the remaining 4 days. At ICU admission, the nutritional target was set for all patients at 20 kcal/kg body weight/day within the first week of ICU admission.

EN was initiated according to current clinical practice guidelines for nutritional support in critically ill patients [13–15]. The 2006 European Society for Clinical Nutrition and Metabolism guidelines on EN in intensive care on page 215 recommend that “during the acute and initial phase of critical illness, an exogenous energy supply in excess of 20–25 kcal/kg body weight/day may be associated with a less favorable outcome, while during the recovery phase, the aim should be to provide 25–30 total kcal/kg body weight/day” [13]. The 2012 Surviving Sepsis Campaign Guidelines suggest avoiding mandatory full caloric feeding in the first week, but they suggest low-dose feeding, advancing only as tolerated (grade 2B) [15]. Therefore, we routinely used a caloric goal of 80–100% of the caloric requirement (25–30 kcal/kg body weight/day after the first week of ICU admission), and only as tolerated the caloric target within the first week of ICU admission (20 kcal/kg body weight/day). The Harris-Benedict equation was also used to determine the caloric target for EN. In addition, the calorie goal for nonobese patients was calculated using the actual body weight, whereas the calorie goal for obese patients (body mass index >30 kg/m^2^) was calculated using the ideal body weight [14]. Supplemental parenteral nutrition (SPN) was added as described in the “Nutrition protocol” section below [16].

EN was administered continuously by the primary care team according to routine protocols that include semirecumbent positioning, preferred use of nasogastric tubes, and the use of prokinetic agents if necessary (gastric residual volume [GRV] ≥200 ml). EN products consisted of polymeric, fiber-enriched formulas containing 1.05–1.62 kcal/ml of energy (18% proteins, 29% lipids [8% medium-chain triglycerides], 53% carbohydrates).

Patients were screened for eligibility within 24 h of ICU admission. The inclusion criteria were (1) >18 years of age, (2) Acute Physiology and Chronic Health Evaluation II (APACHE II) score >8, and (3) expected to stay for at least 24 h in the ICU. The exclusion criteria were (1) AGI could not be evaluated for any reason, (2) advanced cancer, or (3) any terminal stage disease. In addition, the patients were excluded for delayed initiation of EN (>48 h) in the absence of contraindication to EN, evidence for intolerance of EN, or hemodynamic instability. When EN was not feasible, patients at low nutritional risk (Nutritional Risk Screening [NRS-2002] ≤3) who received early SPN (<4 days) or patients at high nutritional risk (NRS-2002 ≥ 5) or who were severely malnourished and received late SPN (>4 days) at admission to the ICU were also excluded from the analyses.

The study protocol was approved by the local ethics committee of each hospital. All patients or their legal representatives provided informed written consent according to the local ethics rules.

### Nutrition protocol

After ICU admission, if the patient had stable hemodynamics or had no EN contraindications, the patient was recommended to receive EN starting 24–48 h after ICU admission. The patients were required to remain in a semirecumbent position. EN was preferentially carried out using a nasogastric tube. The GRV was monitored every 4 h under the condition of indwelling nasogastric tube for a total of six measurements per day. Mean daily GRV was recorded. The EN infusion rate depended upon the total daily infusion of EN. The initial infusion rate was 25 ml/h, and the maximum target infusion rate was determined according to the patient’s feeding tolerance condition and the total daily infusion amount. If the patient reached feeding tolerance of EN (without severe abdominal distention, diarrhea, vomiting, or 4-h GRV <200 ml), then the rate was incrementally increased by 25 ml/h until the EN infusion rate could reach the target (with maximum infusion rate of 100 ml/h). If the GRV of the patients was >200 ml, then intestinal motility drugs were given to improve the feeding tolerance to EN and to strive to reach the calorie target within 48–72 h. After 2-h EN implementation, if GRV was >250 ml, then the initial EN infusion rate was maintained; 2 h later, the GRV was evaluated again, and if the GRV was <250 ml, then the rate was incrementally increased by 25 ml/h until the EN infusion rate could reach the target (with maximum infusion rate of 100 ml/h). If the patient had protein calorie malnutrition at ICU admission and EN could not be implemented, or if EN could not reach 60% of the nutrition target (20 kcal/kg body weight/day within the first week of ICU admission), then the patient was required to receive SPN from the fourth day of ICU admission [16].

### Data collection and definition

A specific case report was used for data collection. Data regarding baseline demographic and clinical characteristics (clinical profile, admission category, presence/absence of sepsis, APACHE II score, Sequential Organ Failure Assessment [SOFA] score, and blood measurements) and nutritional status were collected within the first 24 h of ICU admission. The GI symptoms (vomiting/regurgitation, high GRV, abnormal bowel sounds, diarrhea, bowel distention, and GI bleeding), IAP (minimum, maximum, and mean daily values), and feeding details were documented each day. In addition, the type and amount of nutrition were recorded. Patients who survived were followed by telephone. An unfavorable outcome was defined as 28- and 60-day all-cause mortality after admission to the ICU.

GI symptoms and FI were predefined according to the recommendations of the ESICM [5]. The global AGI grade was determined on the basis of worst AGI grade within the first week of ICU admission and included GI symptoms, IAP, and FI. The IAP was measured via the bladder with patients in the supine position and using the closed-loop system repeated measurements technique. The IAP was measured at least twice daily in the presence of normal values and at least four times daily if IAP was found to be >12 mmHg. FI was considered if at least 20 kcal/kg body weight/day via the enteral route could not be reached within 72 h of a feeding attempt or if EN had to be stopped for any clinical reason (vomiting, high GRV, diarrhea, GI bleeding, or presence of enterocutaneous fistulas) [5]. If EN had to be interrupted for computed tomographic examination, endoscopy, tracheotomy, or any other intervention, then FI was not considered. EN was resumed as soon as possible after the intervention was completed. In the present study, GI bleeding was defined as any bleeding into the GI tract lumen confirmed by macroscopic presence of blood in vomited fluids, gastric aspirate, or stool, according to the recommendations of the ESICM Working Group on Abdominal Problems. GRV was assessed every 4 h after ICU admission for a total of six times per day. GRV monitoring was part of EN feeding. The maximum value within 1 day was assessed, as well as the mean daily value (representing the mean of all the single GRV measurements for 1 day).

### Statistical analysis

SAS 9.13 software (SAS Institute, Cary, NC, USA) was used for database management and statistical analyses. Comparisons of means and proportions relied on the standard normal z-test and Fisher’s exact test, respectively. Continuous variables with a skewed distribution were normalized by logarithmic transformation and presented as geometric mean and 95% CI. Because of the relatively small sample size, AGI was handled as a binary variable as I/II vs. III/IV for the multivariate analyses. The prognostic value of the variables was assessed using a univariate Cox proportional hazards regression model. The variables with *P* values <0.10 were entered in a multivariate Cox proportional hazards regression to determine the independent predictors of all-cause mortality. Covariables in the baseline multivariate regression model included traditional clinical risk factors (age, source of ICU admission, sepsis, diabetes mellitus, coronary artery disease, use of vasoactive drugs, serum creatinine and lactate levels, mechanical ventilation, and APACHE II score). To further evaluate the additional prognostic value of different clinical variables, significant improvements in three different models with the AGI grade on the first day of ICU stay and FI within the first week of ICU stay, respectively, were assessed by the likelihood ratio test, based on the comparison of the χ^2^ value of each model. Kaplan-Meier survival analysis was performed to estimate the cumulative survival. Survival rates of different subgroups of patients stratified on the basis of different AGI grades were compared using the log-rank test. *P* < 0.05 was considered to indicate statistical significance.

## Results

### Characteristics of the patients

**Fig. 1:**
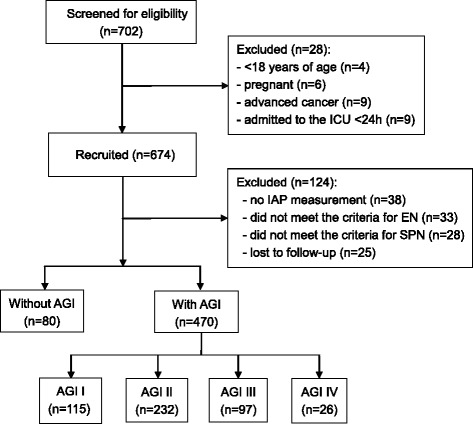
Enrollment flowchart. *AGI* Acute gastrointestinal injury, *EN* Enteral nutrition, *IAP* Intra-abdominal pressure, *ICU* Intensive care unit, *SPN* Supplemental parenteral nutrition

A total of 550 consecutive critically ill patients were recruited from 14 general ICU (69.6% men; mean age, 64.9 ± 17.2 years). Between March 1st, 2014, and April 30th, 2014, 702 patients from 14 ICUs were screened. Among them, 28 cases did not meet the inclusion criteria: 4 patients were <18 years old; 6 patients were pregnant; 9 patients were with advanced tumor; and 9 patients stayed in the ICU <24 h. In addition, 124 patients met the criteria but could not be evaluated for AGI: IAP was not measured in 38 patients; 33 patients received EN outside the guidelines; 28 patients received SPN outside the guidelines; and 25 patients had no follow-up data. Therefore, 550 patients were available for analyses (Fig. 1).

Among the 550 patients, 63.8% were admitted for a medical reason and 27.8% of the patients were admitted for sepsis. The main sources for ICU admission were severe respiratory failure (45.6%), shock (32.8%), and acute kidney injury (18.5%). Among study patients, chronic obstructive pulmonary disease, coronary artery disease, and diabetes mellitus accounted for 18.2%, 16.4%, and 14.7%, respectively. In addition, 456 patients (82.9%) received mechanical ventilation, and 59 (10.7%) received renal replacement therapy (RRT).

### AGI grade and GI symptoms

The 470 patients were evaluated for AGI in the first week of their ICU stay, and the distribution of the global AGI grades was 24.5% for grade I (*n* = 115), 49.4% for grade II (*n* = 232), 20.6% for grade III (*n* = 97), and 5.5% for grade IV (*n* = 26). The mean IAP and mean daily GRV were 9.48 ± 3.37 mmHg and 72.8 ± 58.6 ml, respectively. FI within the first week of ICU stay occurred in 113 patients (24.0%). The mean start time of EN was 30.8 ± 26.2 h. Absent bowel sounds were present in 53.8%, abdominal distention in 44.8%, high GRV in 32.8%, GI bleeding in 25.4%, intra-abdominal hypertension in 39.4%, and abdominal compartment syndrome in 1.9%. Overt GI bleeding associated with compromised hemodynamics occurred in 3.7% of the patients. Using a cutoff value of AGI grade II, the AGI grade on the first ICU day could predict FI occurrence within the first week of ICU stay with 80.2% sensitivity and 65.5% specificity (AUC 0.71, 95% CI 0.64–0.78).

There were no differences among the patients with different grades of AGI for age (*P* = 0.54), sex (*P* = 0.20), source of ICU admission (*P* = 0.38), and related disorders (*P* ≥ 0.20). There were significant differences in systolic and diastolic blood pressures (*P* ≤ 0.01), the use of vasoactive drugs (*P* = 0.012), serum levels of creatinine and lactate (*P* ≤ 0.001), IAP (*P* = 0.001), GRV (*P* = 0.002), calorie intake of EN on the third and seventh days of ICU admission, use of RRT (*P* ≤ 0.001), APACHE II score (*P* = 0.04), and SOFA score (*P* = 0.02) among the different grades of AGI (Table 1).

**Table 1:** Characteristics of the patients according to global acute gastrointestinal injury grade

Characteristics	AGI grade I (*n* = 115)	AGI grade II (*n* = 232)	AGI grade III (*n* = 97)	AGI grade IV (*n* = 26)	*P* value
Age, years	67.7 ± 16.0	64.7 ± 18.3	64.6 ± 18.1	64.4 ± 15.9	0.54
Male sex, *n* (%)	75 (65.2)	157 (67.6)	70 (72.2)	22 (84.6)	0.20
Body mass index, kg/m^2^	21.8 ± 4.04	21.8 ± 2.70	22.2 ± 3.43	21.9 ± 1.95	0.68
NRS 2002	2.13 ± 1.27	2.21 ± 1.20	2.22 ± 1.21	2.62 ± 1.38	0.42
Source of ICU admission					0.38
Medical, *n* (%)	68 (59.1)	149 (64.2)	67(69.1)	18 (69.3)	
Surgical, *n* (%)	21 (18.2)	30 (12.9)	16 (16.5)	5 (19.2)	
Emergency, *n* (%)	26 (22.7)	53 (22.8)	14 (14.4)	3 (11.5)	
Use of vasoactive drug^a^, *n* (%)	30 (26.1)	81 (34.9)	43 (44.3)	14 (53.8)	0.012
Systolic blood pressure^a^, mmHg	114.2 ± 21.5	107.1 ± 22.5	105.7 ± 24.1	97.4 ± 22.1	0.01
Diastolic blood pressure^a^, mmHg	63.0 ± 16.0	57.8 ± 15.4	56.4 ± 12.3	52.2 ± 12.5	0.003
Central venous pressure^a^, mmHg	7.92 ± 2.85	8.33 ± 3.89	8.80 ± 4.23	8.16 ± 2.57	0.60
Heart rate^a^, beats/minute	103.3 ± 24.4	109.6 ± 23.2	115.1 ± 24.6	123.7 ± 26.1	0.002
Hemoglobin^a^, mg/dl	10.7 ± 2.06	10.6 ± 2.74	10.8 ± 2.72	11.9 ± 3.28	0.11
Albumin^a^, mg/dl	32.4 ± 5.78	31.1 ± 6.34	29.6 ± 7.30	31.7 ± 6.96	0.012
Glucose^a^, mmol/L	8.83 ± 3.43	8.98 ± 4.03	9.63 ± 4.82	9.91 ± 4.26	0.28
Serum creatinine^a^, μmol/L	79.4 (56.2–89.1)	88.1 (82.8–93.8)	108.4 (99.5–118.0)	121.6 (94.2–157.1)	<0.0001
Serum lactate^a^, mmol/L	1.59 (1.35–1.87)	1.92 (1.76–2.08)	2.35 (2.27–2.60)	2.41 (1.79–3.24)	0.0004
Related disorders					
Sepsis, *n* (%)	28 (24.3)	63 (27.2)	35 (36.1)	8 (30.8)	0.20
Diabetes mellitus, *n* (%)	20 (17.4)	32 (13.8)	17 (14.4)	5 (19.2)	0.35
Coronary heart disease, *n* (%)	21 (18.3)	29 (12.5)	13 (13.4)	4 (15.4)	0.43
Acute kidney injury, *n* (%)	14 (12.2)	38 (16.4)	30 (30.9)	13 (50.0)	0.005
Renal replacement therapy, *n* (%)	7 (6.09)	18 (7.75)	20 (20.6)	10 (38.4)	<0.001
Mechanical ventilation, *n* (%)	90 (78.3)	196 (84.5)	84 (86.6)	25 (96.2)	0.26
Supplemental parenteral nutrition, *n* (%)	14 (12.2)	40 (17.2)	22 (22.7)	6 (30.8)	0.07
Intra-abdominal pressure^a^, mmHg	8.12 ± 2.52	9.09 ± 2.92	10.3 ± 4.70	10.4 ± 5.09	0.001
Gastric residual volumes^a^, ml	35.0 ± 25.1	56.6 ± 53.6	115.1 ± 95.4	135.0 ± 127.4	0.002
Calorie intake of EN on third day in ICU	1203 ± 192	925 ± 178	792 ± 166	384 ± 152	<0.001
Calorie intake of EN on seventh day in ICU	1449 ± 208	1291 ± 194	825 ± 181	520 ± 165	<0.001
SOFA score^a^	8.0 5 ± 4.62	8.46 ± 3.74	9.32 ± 4.58	11.0 ± 5.22	0.03
APACHE II score^a^	18.3 ± 6.81	19.3 ± 6.84	20.6 ± 7.61	21.9 ± 8.18	0.01
New-onset of infection after admission, *n* (%)	16 (13.9)	55 (23.7)	24 (25.1)	9 (34.6)	0.07
Duration of mechanical ventilation, days	4.37 (3.29–5.80)	6.89 (5.97–7.95)	8.06 (6.95–9.36)	6.63 (4.34–10.1)	0.002
Duration of ICU (day)	8.74 (6.85–11.2)	12.4 (11.0–14.0)	11.9 (10.5–13.3)	8.15 (6.73–12.4)	0.01
28-day mortality, *n* (%)	22 (19.1)	62 (26.7)	42 (43.2)	20 (76.9)	<0.001
60-day mortality, *n* (%)	24 (20.9)	72 (31.0)	46 (47.4)	21 (80.8)	<0.001

*Abbreviations*: *AGI* Acute gastrointestinal injury, *APACHE II* Acute Physiology and Chronic Health Evaluation II, *EN* Enteral nutrition, *ICU* Intensive care unit, *NRS* Nutritional Risk Screening, *SOFA* Sequential Organ Failure Assessment

Values are presented as mean ± SD or as number of subjects (percentage of the column total). *P* values for differences AGI grades were calculated for comparisons on the basis of analysis of variance or Fisher’s exact test (proportions)

^a^Assessed within 24 h of ICU admission

### AGI grading and clinical outcome

The 28- and 60-day mortality rates were 29.3% (*n* = 161) and 32.5% (*n* = 179), respectively. The patients with AGI had higher 28-day (31.1% vs. 18.8%, *P* = 0.025) and 60-day mortality rates (34.7% vs. 20.0%, *P* = 0.01) than those without AGI (Additional file 1: Table S1). In addition, the AGI grade was consistently higher during the 7-day ICU stay among nonsurvivors than among survivors (Additional file 2: Table S2).

The severity of AGI was positively associated with 28- and 60-day mortality. With increasing AGI grade, the patients had higher risk for 28- and 60-day mortality (*P* < 0.001). In addition, the length of ICU stay and duration of mechanical ventilation were significantly (*P* ≤ 0.01) different among patients with different AGI grades (Table 1).

### Univariate and multivariate analyses for 28- and 60-day mortality

Univariate Cox regression analysis showed that age, sepsis, diabetes mellitus, coronary artery disease, the use of vasoactive drugs, serum creatinine and lactate levels, mechanical ventilation, APACHE II score, and global AGI grade were significantly (*P* ≤ 0.02) associated with 60-day mortality. In the multivariate analysis including these variables, diabetes mellitus (HR 1.43, 95% CI 1.03–1.87; *P* = 0.05), the use of vasoactive drugs (HR 1.56, 95% CI 1.12–2.11; *P* = 0.01), serum lactate (HR 1.15, 95% CI 1.06–1.24; *P* = 0.046), global AGI grade (HR 1.65, 95% CI, 1.28–2.12; *P* = 0.008), and APACHE II score (HR 1.04, 95% CI 1.02–1.06; *P* < 0.001) remained independent predictors for 60-day mortality (Table 2). Likewise, similar results were observed for 28-day mortality (data not shown).

**Table 2:** Univariate and multivariate analyses for 60-day mortality in overall patient population

Variables	Univariate analysis			Multivariate analysis		
	HR (95% CI)	χ^2^	*P* value	HR (95% CI)	χ^2^	*P* value
Age (years)	1.01 (1.00–1.02)	6.03	0.01			
Sepsis	1.49 (1.05–2.12)	5.31	0.02			
Diabetes mellitus	1.56 (1.23–2.33)	7.22	0.006	1.43 (1.03–1.87)	3.96	0.05
Coronary heart disease	1.46 (1.04–2.07)	4.69	0.03			
Use of vasoactive drugs	1.91 (1.41–2.55)	13.6	<0.001	1.56 (1.12–2.11)	6.55	0.01
Mechanical ventilation	1.41 (1.04–2.28)	4.58	0.03			
Serum creatinine (μmol/L)	1.002 (1.001–1.003)	13.1	<0.001			
Serum lactate (mmol/L)	1.24 (1.08–1.43)	6.15	0.01	1.15 (1.06–1.24)	4.73	0.03
Global AGI grade (I/II vs. III/IV)	1.78 (1.45–2.13)	11.7	<0.001	1.65 (1.28–2.12)	7.10	0.008
APACHE II score	1.06 (1.04–1.08)	31.0	<0.001	1.04 (1.02–1.06)	12.1	<0.001

*AGI* Acute gastrointestinal injury, *APACHE II* Acute Physiology and Chronic Health Evaluation II

### Added prognostic value of FI

**Fig. 2:**
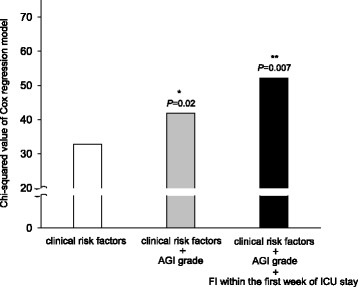
Incremental prognostic value of AGI grade on the first day of ICU admission and FI within the first week of ICU stay in predicting 60-day mortality. Clinical risk factors (age, source of ICU admission, sepsis, diabetes mellitus, coronary artery disease, use of vasoactive drugs, serum creatinine and lactate levels, mechanical ventilation, and APACHE II score) were included in the multivariate regression. The χ^2^ value of each model for predicting all-cause mortality was calculated by the likelihood ratio test. *Significant difference of χ^2^ between the clinical risk factors model and clinical risk factors + AGI grade on the first day of ICU admission model (*P* = 0.02). **Significant difference of χ^2^ between the final model and the clinical risk factors + AGI grade on the first day of ICU admission model (*P* = 0.007). *AGI* Acute gastrointestinal injury, *FI* Feeding intolerance, *ICU* Intensive care unit

In the subgroup analysis that included 402 patients with 7-day survival, three stepwise incremental models including clinical risk factors (age, source of ICU admission, sepsis, diabetes mellitus, coronary artery disease, use of vasoactive drugs, serum creatinine and lactate levels, mechanical ventilation, and APACHE II score), clinical risk factors + AGI grade on the first day of ICU admission, and clinical risk factors + AGI grade on the first day of ICU admission + FI within the first week of ICU stay were used to determine whether FI was associated with adverse outcome. We observed an additional predictive value of 60-day mortality when adding the AGI grade on the first day of ICU admission, with increased χ^2^ value of Cox regression model (χ^2^ = 41.9 vs. 32.8, *P* = 0.02). Furthermore, FI within the first week of ICU stay also provided incremental prognostic value for 60-day mortality in addition to clinical risk factors and AGI grade in the first day of ICU admission, with the highest χ^2^ value (χ^2^ = 52.2 vs. 41.9, *P* = 0.007) (Table 3 and Fig. 2).

**Table 3:** Univariate and multivariate analyses for 60-day mortality in patients with 7-day survival

Variables	Univariate			Multivariate		
	HR (95% CI)	χ^2^	*P* value	HR (95% CI)	χ^2^	*P* value
Age (years)	1.01 (1.00–1.02)	5.77	0.014			
Sepsis	1.31 (1.07–1.89)	4.66	0.04			
Diabetes mellitus	1.37 (1.08–2.01)	5.22	0.02			
Use of vasoactive drugs	1.88 (1.36–2.45)	11.4	<0.001	1.49 (1.23–1.78)	5.93	0.015
Mechanical ventilation	1.33 (1.07–1.95)	4.72	0.04			
Serum creatinine (μmol/L)	1.002 (1.001–1.003)	10.1	<0.001			
Serum lactate (mmol/L)	1.14 (1.02–1.28)	5.39	0.02	1.09 (1.01–1.19)	4.01	0.046
AGI grade on first day of ICU admission (I/II vs. III/IV)	1.83 (1.29–2.42)	10.8	<0.001	1.50 (1.26–1.81)	6.16	0.013
FI within first week of ICU stay	1.75 (1.26–2.38)	9.50	0.002	1.67 (1.22–2.37)	7.32	0.007
APACHE II score	1.05 (1.03–1.08)	25.0	<0.001	1.04 (1.02–1.06)	10.6	0.001

*AGI* Acute gastrointestinal injury, *APACHE II* Acute Physiology and Chronic Health Evaluation II, *FI* Feeding intolerance, *ICU* Intensive care unit

Further analysis indicated that in patients who had survived for 7 days after ICU admission, the caloric intake derived from EN among survivors on the third and seventh days of the ICU stay was significantly higher than that of nonsurvivors (third day, 994 ± 172 vs. 862 ± 176 kcal, *P* = 0.027; seventh day, 1259 ± 183 vs. 1034 ± 190 kcal, *P* = 0.003). The univariate regression analysis showed that the EN intake on the third and seventh days of the ICU stay was significantly associated with the 60-day mortality (HR 0.55, 95% CI 0.37–0.86, *P* = 0.01; and HR 0.50, 95% CI 0.33–0.76, *P* = 0.001, respectively). After adjusting for similar factors, the caloric intake derived from EN on the seventh day of the ICU stay was still independently associated with 60-day mortality (HR 0.59, 95% CI 0.38–0.93; *P* = 0.022), whereas the caloric intake derived from EN on the third day of the ICU stay was not associated with 60-day mortality (HR 0.67, 95% CI 0.43–1.04; *P* = 0.08).

### Kaplan-Meier survival analysis

**Fig. 3:**
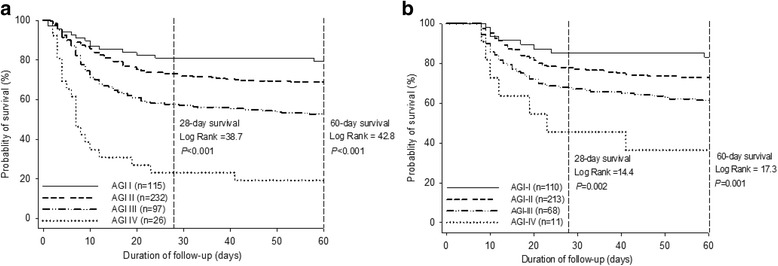
Kaplan-Meier curves stratified on the basis of global AGI grade in the overall population (**a**) and the patients with 7-day survival (**b**) for 28- and 60-day mortality. *P* values were for differences across the AGI grades by log-rank test. *AGI* Acute gastrointestinal injury

Figure 3a shows the Kaplan-Meier curves stratified on the global AGI grade for 28- and 60-day mortality in the overall population. Patients with AGI grades III and IV had 28-day mortality rates of 43.2% and 76.9%, respectively, significantly higher than those with AGI grades I and II (19.1% and 26.7%, respectively; overall χ^2^ = 38.7, *P* < 0.001), whereas there were no significant differences (*P* = 0.18) in 28-day mortality between AGI grades I and II. Similar results were observed for 60-day mortality (overall χ^2^ = 42.8, *P* < 0.001).

**Fig. 4:**
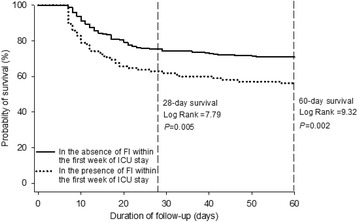
Kaplan-Meier curves stratified on FI within the first week of ICU stay for 28- and 60-day mortality. *P* values were for differences between the patients with and without FI by log-rank test. This analysis included only patients who survived for at least 7 days. *FI* Feeding intolerance, *ICU* Intensive care unit

In the subgroup of 402 patients with 7-day survival, the global AGI grade was found to be significantly associated with 28- and 60-day mortality (overall χ^2^ = 14.4 and 17.3 for 28- and 60-day mortality, respectively; *P* ≤ 0.002) (Fig. 3b). In an analysis stratified by FI within the first week of ICU stay compared with those in the absence of FI, the patients with FI (*n* = 101) had a higher risk of 28-day (37.1% vs. 24.5%; *P* = 0.005) and 60-day mortality (43.8% vs. 29.1%; *P* = 0.002) (Fig. 4).

## Discussion

The main finding of the present study is that the AGI grading system recommended by the ESICM in 2012 could be used to objectively assess GI dysfunction in critically ill patients. In addition, the AGI grade is significantly associated with the risk of mortality. This study provides further evidence supporting the hypothesis that FI within the first week of ICU stay is an independent determinant of mortality beyond its role as a sign of GI dysfunction.

GI dysfunction and its severity were demonstrated to influence the ICU outcome in previous studies [1, 2, 17–19], but the absence of a scaled system for assessing GI function has been a major limiting factor in these studies. Since the introduction of the AGI grading system recommended by the ESICM in 2012, few studies have investigated the association between GI dysfunction stratified using the AGI grading system and clinical outcome. The present study is in line with previous studies in which researchers investigated the association between GI dysfunction or gastrointestinal failure (GIF) assessed by GI symptoms or GIF score and poor outcomes. In their study of a cohort of 377 adult patients from 40 ICUs with expected duration of mechanical ventilation ≥6 h, Reintam et al. reported that GIF (defined as three or more GI symptoms on ICU day 1) was independently associated with a threefold increased risk of mortality. Meanwhile, GIF occurred in 24 patients during the first ICU week and was associated with higher 28-day mortality (62.5% vs. 28.9%, *P* = 0.001) [18]. In a prospective study including 264 patients mechanically ventilated on admission and with an ICU stay >24 h, the mean GIF score based on the combination of FI with IAP during the first 3 days in the ICU was identified as an independent risk factor for ICU mortality (OR 3.02, 95% CI 1.63–5.59; *P* < 0.001) [1]. In addition, the GIF, which was defined as the presence of FI, GI hemorrhage, and/or ileus, has also been indicated to be significantly correlated with an increased risk of mortality, as well as with prolonged ICU stay and mechanical ventilation, in a retrospective analysis of 252 adult patients from 3 ICUs [19].

To our knowledge, the present study is the first to demonstrate that the global AGI grade based on dynamic GI function assessment during the first week of ICU stay, as well as the AGI grade on the first ICU day, was an independent predictor of 60-day mortality. Consistent with the results of our study, a recent study including 196 adult patients with AGI also showed that the AGI grading system could reflect the severity of illness, and the dichotomization of AGI into two grades (AGI I + II vs. III + IV) appeared to have prognostic value [20]. Taken together, these findings support the concept that GI dysfunction not only indicates a critical condition but also is an important determinant in the clinical outcome of patients admitted to the ICU.

FI is a general term indicating the interruption of enteral feeding for any clinical reason, and it is inconsistently defined. FI is considered a sign of GI dysfunction. Despite the problems with definition, current evidence supports the concept that FI occurs frequently and is associated with adverse outcomes in critically ill patients. In an international observational cohort study of 1888 ICU patients, the frequency of FI (defined as GRV ≥200 ml) was 30.5% and was associated with worse nutrition adequacy (56% vs. 64%, *P* < 0.0001), increased ICU stay (14.4 vs. 11.3 days, *P* < 0.0001), and increased mortality (30.8% vs. 26.2%, *P* = 0.04) [8]. In a retrospective observational study, Reintam et al. observed that the frequency of FI and the association between FI and mortality varied widely and depended on the definition used. Of these various definitions, the definition of FI based on the presence of at least three of five GI symptoms was the most strongly related to ICU mortality, whereas EN <23% of the caloric target had the strongest predictive power for 90-day mortality [19]. In a recent meta-analysis (5 studies and 897 patients), FI was shown to be associated with increased mortality and prolonged ICU stay [6]. In the present study, it was considered that persistent FI within the first week of ICU stay was an independent predictor of 60-day mortality on the basis of clinical risk factors and AGI grading of the first ICU day.

In view of AGI grading on the first ICU day as assessed by a combination of GI symptoms and IAP, the present study indicated that FI was not completely correlated to severity of GI dysfunction. The combination of GI symptoms and persistent enteral underfeeding within the first ICU week could improve risk stratification in critically ill patients. Nevertheless, the exact role of FI regarding adverse outcomes remained to be clarified. Although the optimal caloric intake of EN for critically ill patients remains controversial [21–24], the present study suggests that FI leads to unfavorable outcomes. In addition, we observed that the lower EN intake within the first week of ICU stay was significantly associated with an increased mortality. Our results suggest that in addition to GI dysfunction itself, resultant underfeeding (FI induced by GI dysfunction and its related results) could further aggravate the adverse prognosis.

This study should be interpreted within the context of its strengths and limitations. We conducted a multicenter, prospective, observational study with a relatively large sample size. Furthermore, the AGI grade was assessed daily during the first week of ICU stay and could dynamically reflect the change of GI dysfunction. Nevertheless, the present study has several limitations. First, the AGI grading system lacks objective measures for GI function/dysfunction. In addition, FI was determined on the basis of failure to achieve EN caloric targets, which are the key issues currently limiting the research in this area. The target goal of EN set at 20 kcal/kg body weight/day as a principle of permissive underfeeding in the first week of ICU admission could potentially lead to a decreased incidence of FI as well as high GRV. Second, only patients with prolonged ICU stay (>24 h) were enrolled, which could bias the results. Third, the inclusion of a relatively large number of patients with 7-day survival limits the generalizability of these results. Fourth, the use of prokinetics based on GRV ≤200 ml might reduce the incidence of FI and severity of AGI grade. Finally, the multivariate model included baseline variables as well as variables assessed within 1 week of admission, and some of them could be collinear. In addition, there could be some collinearity between AGI at admission and the occurrence of FI over the first 7 days.

## Conclusions

AGI grading is a strong predictor for mortality. FI within the first week of ICU stay has an independent and incremental prognostic value for mortality, suggesting that the combination of the AGI grade on the first day of ICU admission and persistent FI within the first week of ICU stay could improve risk stratification in critically ill patients.

## Additional files

Additional file 1: Table S1. Characteristics of the patients with and without AGI. (DOC 54 kb)

Additional file 2: Table S2. AGI grading during the 7-day ICU stay among survivors and nonsurvivors. (DOC 61 kb)

## Abbreviations

AGI: Acute gastrointestinal injury
APACHE II: Acute Physiology and Chronic Health Evaluation II
EN: Enteral nutrition
ESICM: European Society of Intensive Care Medicine
FI: Feeding intolerance
GI: Gastrointestinal
GIF: Gastrointestinal failure
GRV: Gastric residual volume
IAP: Intra-abdominal pressure
ICU: Intensive care unit
MOF: Multiple organ failure
NRS: Nutritional Risk Screening
RRT: Renal replacement therapy
SOFA: Sequential Organ Failure Assessment
SPN: Supplemental parenteral nutrition
