# Supplementary material for: Severity of acute gastrointestinal injury grade is a predictor of all-cause mortality in critically ill patients: a multicenter, prospective, observational study
Source: Crit Care. 2017 Jul 14;21:188. doi: 10.1186/s13054-017-1780-4 (PMC5513140; doi:10.1186/s13054-017-1780-4)
Supplement: Supplementary file 1 — Characteristics of the patients with and without AGI. (DOC 54 kb) [file 13054_2017_1780_MOESM1_ESM.doc]

**Table S1.** Characteristics of the patients with and without AGI

| Characteristics | Non-AGI (n=80) | AGI (n=470) | *P* |
| --- | --- | --- | --- |
| Age (years) | 65.8 ± 16.8 | 65.1 ± 17.8 | 0.73 |
| Male, n (%) | 55 (68.7) | 324 (68.9) | 0.97 |
| Source of ICU admission |  |  | 0.84 |
| Medical, n (%) | 49 (61.3) | 302 (64.2) |  |
| Surgical, n (%) | 14 (17.5) | 72 (15.3) |  |
| Emergency, n (%) | 17 (21.3) | 96 (20.4) |  |
| Use of vasoactive drug, n (%) | 19 (23.8) | 168 (35.7) | 0.03 |
| Systolic blood pressure (mmHg)* | 116.0 ± 25.5 | 105.2 ± 23.4 | 0.0002 |
| Diastolic blood pressure (mmHg)* | 62.6 ± 16.5 | 56.3 ± 14.1 | 0.0004 |
| Central venous pressure (mmHg)* | 7.89 ± 3.67 | 8.55 ± 3.92 | 0.32 |
| Heart rate (beat/min)* | 105.0 ± 24.7 | 112.4 ± 24.1 | 0.007 |
| Hemoglobin (mg/dl)* | 10.7 ± 2.28 | 10.8 ± 2.76 | 0.79 |
| Albumin (mg/dl)* | 33.2 ± 7.25 | 30.5 ± 6.79 | 0.001 |
| Glucose (mmol/L)* | 8.44 ± 3.70 | 9.27 ± 4.16 | 0.10 |
| Serum creatinine (μmol/L)* | 76.2 (66.1- 87.2) | 95.0 (90.2 - 100.1) | <0.001 |
| Serum lactate (mmol/L)* | 1.55 (1.41-1.70) | 2.13 (1.99 - 2.28) | 0.005 |
| Related disorders |  |  |  |
| Sepsis, n (%) | 19 (23.8) | 134 (28.5) | 0.38 |
| Diabetes mellitus, n (%) | 12 (15.0) | 74 (16.8) | 0.86 |
| Coronary heart disease, n (%) | 14 (17.5) | 67 (14.3) | 0.45 |
| Acute kidney injury, n (%) | 6 (7.50) | 95 (20.2) | 0.007 |
| Renal replacement therapy, n (%) | 3 (3.75) | 55 (11.7) | 0.03 |
| Mechanical ventilation, n (%) | 61 (76.2) | 395 (84.0) | 0.09 |
| Intra-abdominal pressure (mmHg)* | 6.88 ± 2.45 | 9.79 ± 3.74 | <0.001 |
| Gastric residual volumes (ml)* | 28.5 ± 19.1 | 86.9 ± 75.6 | 0.002 |
| SOFA score* | 7.74 ± 3.81 | 9.08 ± 4.30 | 0.014 |
| APACHE II score* | 16.6 ± 6.26 | 19.4 ± 6.72 | 0.001 |
| New-onset of infection after admission, n (%) | 12 (15.0) | 104 (22.1) | 0.15 |
| Duration of mechanical ventilation (days) | 4.41 (3.18 - 5.92) | 7.23 (6.14 - 8.48) | 0.01 |
| Duration of ICU (days) | 8.85 (6.95 - 11.4) | 11.2 (9.9 - 12.6) | 0.03 |
| 28-day mortality, n (%) | 15 (18.8) | 146 (31.1) | 0.025 |
| 60-day mortality, n (%) | 16 (20.0) | 163 (34.7) | 0.01 |

Values are presented as mean±SD, or as number of subjects (percentage of the column total). *P* values for differences AGI grades were calculated for comparisons on the basis of analysis of variance or Fisher’s exact test (proportions). NRS: Nutrition Risk Screening 2002; *Assessed within 24 h of ICU admission.

**Table S1** shows the characteristics of the patients with and without AGI. The patients with and without AGI had similar age (*P*=0.73), gender (*P*=0.97), and the source of ICU admission (*P*=0.84). However, The 470 patients with AGI, compared to those with non-AGI (n=80), had higher serum creatinine and lactate (*P*≤0.005), intra-abdominal pressure (*P*<0.001), gastric residual volumes (*P*=0.002), duration of ICU stay (*P*=0.03), APACHE II score (*P*=0.001), the prevalence of acute kidney injury (*P*=0.007), and 28- and 60-day mortality rates (*P*≤0.025), but lower systolic and diastolic blood pressures (*P*≤0.0004), and plasma albumin level (*P*=0.001).
