# Supplementary material for: Severity of acute gastrointestinal injury grade is a predictor of all-cause mortality in critically ill patients: a multicenter, prospective, observational study
Source: Crit Care. 2017 Jul 14;21:188. doi: 10.1186/s13054-017-1780-4 (PMC5513140; doi:10.1186/s13054-017-1780-4)
Supplement: Supplementary file 2 — AGI grading during the 7-day ICU stay among survivors and nonsurvivors. (DOC 61 kb) [file 13054_2017_1780_MOESM2_ESM.doc]

**Table S2.** AGI grading during the 7-day ICU stay among survivors and non-survivors.

| AGI Grade | Survivors  (n=371) | Non-survivors  (n=179) | *P* |
| --- | --- | --- | --- |
| AGI grade on the 1^st^ day of ICU stay |  |  | <0.001 |
| Without AGI, n (%) | 77 (20.8) | 20 (11.2) |  |
| AGI I, n (%) | 106 (28.6) | 31 (17.3) |  |
| AGI II, n (%) | 140 (37.7) | 70 (39.1) |  |
| AGI III, n (%) | 45 (12.1) | 41 (22.9) |  |
| AGI IV, n (%) | 3 (0.8) | 17 (9.5) |  |
| AGI grade on the 2^nd^ day of ICU stay |  |  | <0.001 |
| Without AGI, n (%) | 72 (19.4) | 18 (10.1) |  |
| AGI I, n (%) | 99 (26.7) | 29 (16.2) |  |
| AGI II, n (%) | 149 (40.2) | 72 (40.2) |  |
| AGI III, n (%) | 48 (12.9) | 43 (24.0) |  |
| AGI IV, n (%) | 3 (0.8) | 17 (9.5) |  |
| AGI grade on the 3^rd^ day of ICU stay |  |  | <0.001 |
| Without AGI, n (%) | 65 (17.5) | 18 (10.1) |  |
| AGI I, n (%) | 94 (25.3) | 27 (15.1) |  |
| AGI II, n (%) | 156 (42.0) | 73 (40.8) |  |
| AGI III, n (%) | 51 (13.7) | 43 (24.0) |  |
| AGI IV, n (%) | 5 (1.3) | 18 (10.1) |  |
| AGI grade on the 4^th^ day of ICU stay |  |  | <0.001 |
| Without AGI, n (%) | 64 (17.3) | 17 (9.5) |  |
| AGI I, n (%) | 91 (24.5) | 25 (14.0) |  |
| AGI II, n (%) | 160 (43.1) | 76 (42.5) |  |
| AGI III, n (%) | 51 (13.7) | 42 (23.5) |  |
| AGI IV, n (%) | 5 (1.3) | 19 (10.6) |  |
| AGI grade on the 5^th^ day of ICU stay |  |  | <0.001 |
| Without AGI, n (%) | 64 (17.3) | 16 (8.9) |  |
| AGI I, n (%) | 97 (26.1) | 25 (14.0) |  |
| AGI II, n (%) | 157 (42.3) | 73 (40.8) |  |
| AGI III, n (%) | 49 (13.2) | 44 (24.6) |  |
| AGI IV, n (%) | 4 (1.1) | 21 (11.7) |  |
| AGI grade on the 6^th^ day of ICU stay |  |  | <0.001 |
| Without AGI, n (%) | 67 (18.1) | 16 (8.9) |  |
| AGI I, n (%) | 102 (27.5) | 24 (13.4) |  |
| AGI II, n (%) | 155 (41.8) | 72 (40.2) |  |
| AGI III, n (%) | 43 (11.6) | 46 (25.7) |  |
| AGI IV, n (%) | 4 (1.1) | 21 (11.7) |  |
| AGI grade on the 7^th^ day of ICU stay |  |  | <0.001 |
| Without AGI, n (%) | 71 (19.1) | 16 (8.9) |  |
| AGI I, n (%) | 109 (29.4) | 24 (13.4) |  |
| AGI II, n (%) | 151 (40.7) | 72 (40.2) |  |
| AGI III, n (%) | 37 (10.0) | 46 (25.7) |  |
| AGI IV, n (%) | 3 (0.8) | 21 (11.7) |  |

**Table S2** shows the changes of AGI grading during the 7-day ICU stay among survivors and non-survivors. The AGI grade was consistently higher during the 7-day ICU stay among non-survivors compared with survivors. In addition, there was an increased prevalence of AGI among survivors and non-survivors as time went on during the 7-day ICU stay.
